# The Development of the Gut Microbiota and Short-Chain Fatty Acids of Layer Chickens in Different Growth Periods

**DOI:** 10.3389/fvets.2021.666535

**Published:** 2021-07-02

**Authors:** Baosheng Sun, Linyue Hou, Yu Yang

**Affiliations:** Laboratory of Poultry Production, College of Animal Science, Shanxi Agricultural University, Jingzhong, China

**Keywords:** gut microbiota, chicken, period, short-chain acid, functional prediction

## Abstract

A long-term observation of changes of the gut microbiota and its metabolites would be beneficial to improving the production performance of chickens. Given this, 1-day-old chickens were chosen in this study, with the aim of observing the development of the gut microbiota and gut microbial function using 16S rRNA gene sequencing and metabolites short-chain fatty acids (SCFAs) from 8 to 50 weeks. The results showed that the relative abundances of Firmicutes and genus *Alistipes* were higher and fiber-degradation bacteria were less at 8 weeks compared with 20 and 50 weeks (*P* < 0.05). Consistently, gut microbial function was enriched in ATP-binding cassette transporters, the energy metabolism pathway, and amino acid metabolism pathway at 8 weeks. In contrast, the abundance of Bacteroidetes and some SCFA-producing bacteria and fiber-degradation bacteria significantly increased at 20 and 50 weeks compared with 8 weeks (*P* < 0.05), and the two-component system, glycoside hydrolase and carbohydrate metabolism pathway, was significantly increased with age. The concentration of SCFAs in the cecum at 20 weeks was higher than at 8 weeks (*P* < 0.01), because the level of fiber and the number of dominant fiber-degradation bacteria and SCFA-producing bacteria were more those at 20 weeks. Notably, although operational taxonomic units (OTUs) and the gut microbial α-diversity including Chao1 and abundance-based coverage estimator (ACE) were higher at 50 than 20 weeks (*P* < 0.01), the concentration of SCFAs at 50 weeks was lower than at 20 weeks (*P* < 0.01), suggesting that an overly high level of microbial diversity may not be beneficial to the production of SCFAs.

## Introduction

The gut microbiota play an important role in the poultry nutrition and health (1). Age is a dramatic factor that affects the microbial communities. The gut microbiota are distinct in different growth periods of chickens (2, 3). In the initial stage of colonization, facultative anaerobes were the principal bacteria, followed by strict anaerobes (4). *Bacteroides* and *Eubacteria* are established in 2 weeks, and the gut microbiota take 6–7 weeks to complete their establishment in chickens (5). The predominant phyla in the cecum are Firmicutes and Bacteroidetes over the whole life of chickens (6). Most of the current research on the succession of bacteria of chickens with age has focused on broilers (7–10). It was found that the colonization and function of the gut microbiota in broilers were different from 1 to 42 days (11, 12). In addition, some studies have focused on the microbiota of layer hens (13–16), the majority of which explored the short-term effect on the microbiota of young layers. In term of long-term observation, the gut microbiota in commercial Hy-Line layers from 1 to 51 weeks under field conditions were observed (17). A long-term development of cecal microbiota in egg-laying hens Lohmann Brown Light chickens from the day of hatching to 60 weeks old was also characterized (18). However, the above research on the layers' microbiome rarely observed the changes of intestinal microbiota function and microbial metabolites such as short-chain fatty acids (SCFAs) at the same time with age.

SCFAs, mainly including acetate, propionate, and butyrate, are derived from bacterial degradation and fermentation of dietary fibers. The cecum is the principal place for microbial fermentation of dietary fiber in chickens. Bacteroidetes is a kind of “generalist” that degrades dietary fiber polysaccharides. It can utilize a wide range of dietary polysaccharides from plants (19). Excellent fiber-degrading members of Bacteroidetes including *Bacteroides* (20) and *Prevotella* (21). *Ruminococcus, Fibrobacter* (22), *Clostridium*, and *Roseburia* (23) are excellent cellulolytic members of Firmicutes. SCFAs contribute to host nutrition and immune health (15). SCFAs can be used as energy and carbon source for poultry (24, 25). Acetate enters the liver for metabolism as a substrate for peripheral adipogenesis. Propionate reaches the liver as a substrate for gluconeogenesis. Butyrate serves as an energy source for colonic epithelial cells once SCFAs are absorbed. SCFAs can also regulate metabolism by inhibiting histone deacetylase (HDAC) and G protein-coupled receptors (GPCRs), such as GPR41 or GPR43 (26). In addition, SCFAs reduce intestinal pH (27) and induce the differentiation of regulatory T cells (28) to enhance the host health. SCFA production was also impacted by age (29).

Research on longitudinal observation of the ISA Brown layers' microbiome and SCFAs is lacking. Given this, ISA Brown Hens (IBH) were chosen in this experiment to observe the succession of the gut microbiota, enriched metabolic pathways, and SCFAs in different growth periods. Increasing our understanding of this would be beneficial to promoting production performance and the health of chickens by improving the gut microbiota and SCFAs.

## Materials and Methods

### Experimental Design and Animal Management

This study was approved by the Shanxi Agricultural University Animal Experiment Ethics Committee (the license number: SXAU-EAW-2017-002Chi.001). In total, 108 1-day-old IBH were chosen. Chickens were randomly divided into nine replicates, with 12 chickens per replicate. Chickens were fed three different diets (Table 1) during brooding periods (0–8 weeks), growing periods (9–20 weeks), and laying periods (21–50 weeks).

**Table 1:** Ingredients and nutrient levels of diets used in different growth periods.

**Item**	**0–8 weeks**	**9–20 weeks**	**21–50 weeks**
**Ingredients (%)**			
Corn	61.95	60.49	60.00
Soybean meal	23.7	10	15.5
Bran	0	8.5	0
Soybean oil	1.1	0.5	0.6
Corn gluten meal	4	0	1.6
Spray corn husk	0	6.5	3.5
DDGS	4	5.75	5
Peanut meal	0	0	1
Stone power	1.8	2.1	9.02
CaHPO_4_	1.3	0.7	0.65
NaCI	0.3	0.28	0.25
Met	0.2	0.06	0.14
Lys	0.46	0.08	0.19
Thr	0.09	0.04	0
Multivitamin^a^	0.4	0.35	0.4
Minerals^b^	0.55	0.5	0.45
Zeolite	0	2	0.5
Choline chloride	0.1	0.05	0.1
Complex enzyme	0.05	0	0
Monosodium glutamate	0	2	1
Protein powder	0	0.1	0.1
Total	100	100	100
**Nutrient levels*^c^***			
ME (MJ/kg)	12.43	11.50	10.62
Crude protein (%)	19.49	15.3	16
Crude fiber (%)	3.21	3.95	2.95
Crude fat (%)	4.27	3.99	3.72
Crude ash (%)	5.83	5.67	12.13
Ca (%)	1.05	0.99	3.55
Total P (%)	0.57	0.5	0.43
NaCI (%)	0.3	0.37	0.31

*DDGS, dried distiller's grain with solubles*.

a *0–8 weeks: per kilogram of diet contained vitamin A 2,100–2,500 KIU; vitamin B1 ≥ 620 mg; vitamin B2 ≥ 1,600 mg; vitamin B5 ≥ 2,450 mg; vitamin B6 ≥ 830 mg; niacinamide ≥ 7,000 mg; vitamin B12 ≥ 4,200 μg; vitamin D3 800–1,240 KIU; vitamin E ≥ 5,900 IU; vitamin K3 ≥ 600 mg; folic acid ≥ 245 mg; biotin ≥ 35 mg. 9–20 weeks: vitamin A 2,150–3,250 KIU; vitamin B1 ≥ 440 mg; vitamin B2 ≥ 1,280 mg; vitamin B5 ≥ 2,140 mg; vitamin B6 ≥ 640 mg; vitamin B12 ≥ 3,200 mg; vitamin D3 550–1,650 KIU; vitamin E ≥ 4,260 IU; vitamin K3 ≥ 430 mg; nicotinamide ≥ 6,400 mg; folic acid ≥ 280 mg; biotin ≥ 40 mg. 21–50 weeks: vitamin A 145 to 190 KIU; vitamin B1 ≥ 26 mg; vitamin B2 ≥ 100 mg; vitamin B5 ≥ 160 mg; vitamin B6 ≥ 60 mg; vitamin B12 ≥ 200 mg; vitamin D3 30 to 95 KIU; vitamin E ≥ 355 IU; vitamin K3 ≥ 50 mg; nicotinamide ≥ 552 mg; folic acid ≥ 12 mg; biotin ≥ 2 mg; choline chloride ≥ 6.5 mg*.

b *0–8 weeks: per kilogram of diet contained Cu 8 mg, Fe 80 mg, Me 60 mg, Se 0.15 mg, Zn 40 mg, I 0.35 mg. 9–20 weeks: Fe 1,300–7,400 mg; Cu 120–650 mg; Mn 1,450–2,900 mg; Zn 1,250–2,900 mg; I 7–95 mg; Se 6–9.5%; Ca 12–25%; P (adding phytase) ≥ 2.0%; NaCI 4–10%; methionine ≥ 1.8%; moisture ≤ 10%. 21–50 weeks: Fe 1,300–7,400 mg; Cu 120–650 mg; Mn 1,450–2,900 mg; Zn 1,250–2,900 mg; I 8–95 mg; Se 6–9.5%; Ca 12–25%; P (adding phytase) ≥ 2.1%; NaCI 4–10%; methionine ≥ 2.3%; moisture ≤ 10%*.

c *The composition was calculated but not measured for the diet used*.

Chickens were given free access to water and diet. The management of the temperature, light, and humidity was conducted according to the breeding manual. No conventional immunization schedule was performed to avoid impacts on the gut microbiota.

### Sample Collection

A chicken from each replicate was chosen at the end of 8, 20, and 50 weeks. They were slaughtered humanely using oral bloodletting slaughtering method. The contents of the left cecum per bird were collected into multiple cryogenic tubes, and they were put into a liquid nitrogen tank and then preserved at −80°C until the 16S rRNA gene sequence of the gut microbiota and the determination of the concentration of SCFAs.

### Sample Determination

#### 16S rRNA Gene Sequencing of Gut Microbiota

It was sequenced by Genedenovo Biotechnology Ltd. (Guangzhou, China) using High-Throughput Sequencing Technology. First, DNA extraction was performed using the HiPure Stool DNA Kits (Guangzhou, China). V3–V4 regions of the 16S rRNA gene were amplified by PCR using primers 341F 5′-CCTACGGGNGGCWGCAG and 806R 3′-GGACTACHVGGGTATCTAAT. The barcode is an eight-base sequence unique to each sample. PCRs were as follows: 95°C for 2 min, followed by 27 cycles at 98°C for 10 s, 62°C for 30 s, 68°C for 30 s, and a final extension at 68°C for 10 min. PCRs were performed in triplicate with 50 μl of mixture containing 5 μl of 10 × KOD Buffer, 5 μl of 2.5 mM of dNTPs, 1.5 μl of each primer, 1 μl of KOD Polymerase, and 100 ng of template DNA.

Illumina Hiseq 2500 (Illumina, Inc., San Diego, CA, USA) sequencing was then performed. Amplicons were extracted from 2% agarose gels and purified using the AxyPrep DNA Gel Extraction Kit (Axygen Biosciences, Union City, CA, USA) and quantified using ABI Ste1OnePlus Real-Time PCR System (Life Technologies, Carlsbad, CA, USA). Purified amplicons were pooled in equimolar and paired-end sequenced (2 ×250) on an Illumina platform. The datasets presented in this study can be found in online repositories. The names of the repository and accession number can be found in the National Center for Biotechnology Information (NCBI) Sequence Read Archive (SRA), PRJNA701972.

#### Bioinformatics Analysis

##### Quality Control and Read Assembly

Raw reads were further filtered using FASTP. Paired-end clean reads were merged as raw tags using FLSAH (30) (version 1.2.11) with a minimum overlap of 10 bp and mismatch error rates of 2%. Raw tag filtering noisy sequences of raw tags were filtered by QIIME (31) (version 1.9.1) pipeline under specific filtering conditions (32) to obtain the high-quality clean tags. *Chimera checking and removal*: Clean tags were searched against the reference database to perform reference-based chimera checking using UCHIME algorithm. All chimeric tags were removed, and the final obtained effective tags were used for further analysis.

##### Operational Taxonomic Unit Cluster

Effective tags were clustered into operational taxonomic units (OTUs) with 97% similarity using the UPARSE pipeline (33). The tag sequence with the highest abundance was selected as a representative sequence within each cluster. Venn analysis was performed in R project (version 3.4.1) to identify unique and common OTUs.

##### Taxonomy Classification

The representative sequences were classified into organisms using the Ribosomal Database Project classifier (version 2.2) (34) based on SILVA database (35) with the confidence threshold values ranging from 0.8 to 1. The abundance statistics of each taxonomy were visualized using Krona (36) (version 2.6).

##### Microbial Diversity Analysis

α-Diversity indices including abundance-based coverage estimator (ACE), Chao1, Shannon, and Simpson were calculated in QIIME. The comparison of α-diversity indices among groups was performed by the Kruskal–Wallis using Vegan package in R project (37). β-Diversity was performed. Sequence alignment was performed using Muscle (38) (version 3.8.31), and then weighted uniFrac distance matrix was generated by GuniFrac package (version 1.0) in R project.

##### Function Prediction

Functional profiles including Kyoto Encyclopedia of Genes and Genomes (KEGG) Orthology (KO) and enriched metabolism pathways of OTUs were inferred using a software package Tax4Fun (39). Microbiome phenotypes of bacteria were classified using BugBase. FAPROTAX database (Functional Annotation of Prokaryotic Taxa) and associated software (version 1.0) were used for generating the ecological functional profiles of bacteria. Heatmaps were made by R pheatmap package. The predicted KO and ko abundances were normalized by *Z*-score and then plotted.

#### Determination of the Concentration of Short-Chain Fatty Acids

The concentration of SCFAs (mmol/100 g) in the cecum chyme was measured using the internal standard method with High Performance Gas Chromatography (Trace 1300, Thermo Fisher Scientific, Waltham, MA, USA) (40).

First, a solution containing internal standard crotonic acid was prepared. Metaphosphoric acid 25 g and crotonic acid 0.6464 g were accurately weighed, and they were put into a 100-ml volumetric flask and up to 100 ml with ultrapure water. Then, 100 ml of mixed standard stock solutions was prepared as follows: different volumes of standards were added (Table 2) into a 100-ml volumetric flask, topped up to 100 ml with ultrapure water, and preserved at 4°C. The concentration (g/L) of additive was calculated according to the density of each standard (e.g., acetate is 1.050 g/ml), and then it was converted into the mol concentration (mmol/L) based on molar mass of each standard (e.g., acetate is 60 mol/g). The volatile fatty acid standard solution was prepared as follows: 0.2 ml of deproteinized metaphosphate solution containing crotonic acid was added to three 1.5-ml centrifuge tubes, and 1 ml of mixed standard stock solution was added to this. The peak area of crotonic acid in the standard solution was measured.

**Table 2:** The additive volume and concentrations of volatile fatty acid standards added to the standard stored solution.

	**Acetate**	**Propionate**	**Butyrate**	**Isobutyrate**	**Isovalerate**	**Valerate**
Additive volume(μl)	60	40	20	5	5	5
Concentration^a^(g/L)	0.63	0.40	0.19	0.048	0.047	0.047
Mol concentration^b^(mmol/L)	10.50	5.35	2.19	0.54	0.46	0.46

a *Concentration of additive standards (g/L) = density of standards (g/mL) × additive volume (μl) ÷ 100*.

b *Mol concentration (mmol/L) = concentration of additive standard (g/L) ÷ molar mass of standard (g/mol) ×1,000*.

*Sample preparation*: 0.5- to 1-g contents of the cecum were added to nine times the weight of ultrapure water, homogenate, and centrifuged at 10,000 rpm for 10 min, and the supernatant was removed. Then, 1 ml of supernatant sample was placed into a 1.5-ml Eppendorf (EP) tube, and 0.2 ml of mixed solution of crotonic metaphosphate was added and reacted for 3 h. Centrifugation at 12,000 r for 5 min was undertaken. The supernatant was injected into the chromatograph instantaneously with a 10-μl microinjector, and the injection volume was 1.0 μl. Reaction conditions were set as follows: injection temperature 220°C; initial temperature 70°C; detector temperature 220°C; split 5; split ratio 6; constant current 0.8 ml/min; tail blowing 40 ml/min; and hydrogen 35 ml/min and air 350 ml/min.

The concentration of a certain acid (mmol/L) = (peak area of certain acid of sample × peak area of crotonic acid in standard solution × mol concentration of certain acid) ÷ (peak area of crotonic acid in sample × peak area of certain acid in standard solution).

### Statistical Analysis

In terms of gut microbiota, the comparisons of the relative abundance phyla and genera in groups were performed by Metastats (41) (version 20090414). Metastats showed significantly different relative abundances of bacteria using *P* < 0.01 or 0.05. Multivariate statistical techniques including principal component analysis (PCA), principal coordinates analysis (PCoA), and non-metric multidimensional scaling (NMDS) of weighted uniFrac distances were calculated and plotted in R project. Statistical analysis of Welch's *t*-test and Anosim test was calculated using R project. The β-diversity analyses between groups were calculated by the Kruskal–Wallis using Vegan package in R project. Heatmap analysis was performed using the R package. Analysis of function difference between groups was calculated by Welch's *t*-test in R package (version 2.5.3). Statistical analyses of SCFAs were performed using a one-way ANOVA with SPSS 22.0 software. The results are expressed as the means and standard error of the mean (SEM).

## Results

### Operational Taxonomic Units and Microbial Diversity of Chickens in Different Periods

For simplicity, “ISA Brown Hens-8 weeks” was named IBHE for short, “ISA Brown Hens-20 weeks” was named IBHT, and “ISA Brown Hens-50 weeks” was named IBHF.

**Figure 1:**
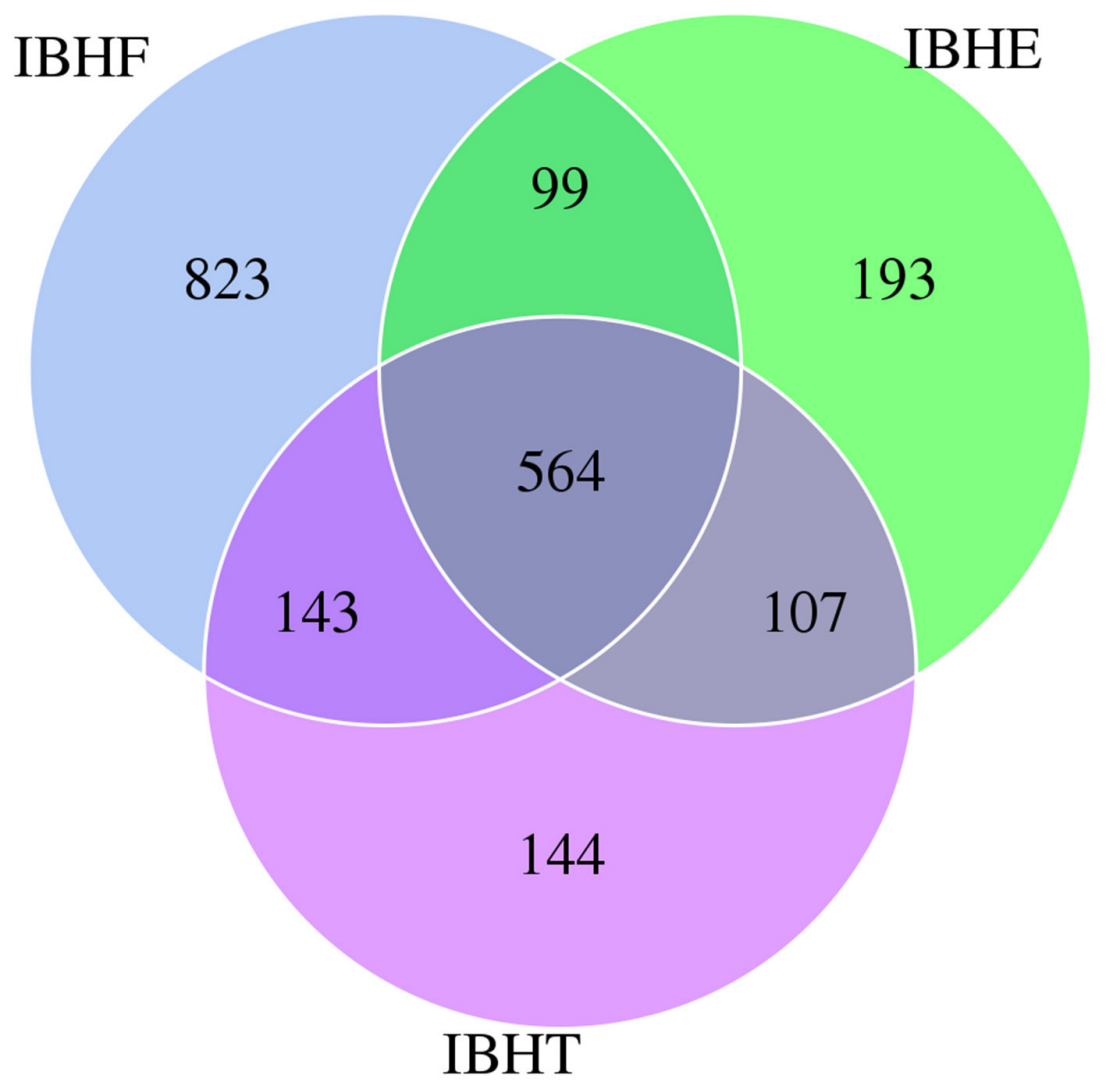
Venn diagram of operational taxonomic units (OTUs) in different growth periods. Note that the overlapping parts show the OTUs shared by three groups, and the numbers on both sides are the unique OTUs owned by each group. IBHE, IBHT, and IBHF refer to “ISA Brown Hens-8 weeks,” “ISA Brown Hens-20 weeks,” and “ISA Brown Hens-50 weeks,” respectively.

The total and unique numbers of OTUs at 50 weeks (1,629 and 823) were more than those at 8 (963 and 193) and 20 weeks (958 and 144) (Figure 1). Moreover, the α-diversity indices including ACE and Chao1 significantly increased with age (*P* < 0.01) (Table 3). It is suggested that the microbial community richness significantly increased with age. The gut microbial diversity of IBHF was the highest among three periods (Table 3); this was also consistent with the number of OTUs.

**Table 3:** Comparison of α-diversity indices among different growth periods.

**Diversity indices**	**8 weeks**	**20 weeks**	**50 weeks**	**SEM**	***P*-value**
ACE	1,594.39^Bc^	1,653.17^Bb^	2,460.21^Aa^	168.13	0.0010
Chao1	1,547.78^Bc^	1,660.92^Bb^	2,422.61^Aa^	134.25	0.0070
Shannon	6.54	6.51	7.16	0.26	0.017
Simpson	0.95	0.97	0.98	0.0047	0.56

*Means within a row lacking a common lowercase superscript letter mean significant differences with a P-value < 0.05, and means within a row lacking a common uppercase superscript letter mean extremely significant differences with a P-value < 0.01. Data are expressed as the means and pooled standard error of the mean (SEM)*.

*ACE, abundance-based coverage estimator*.

**Figure 2:**
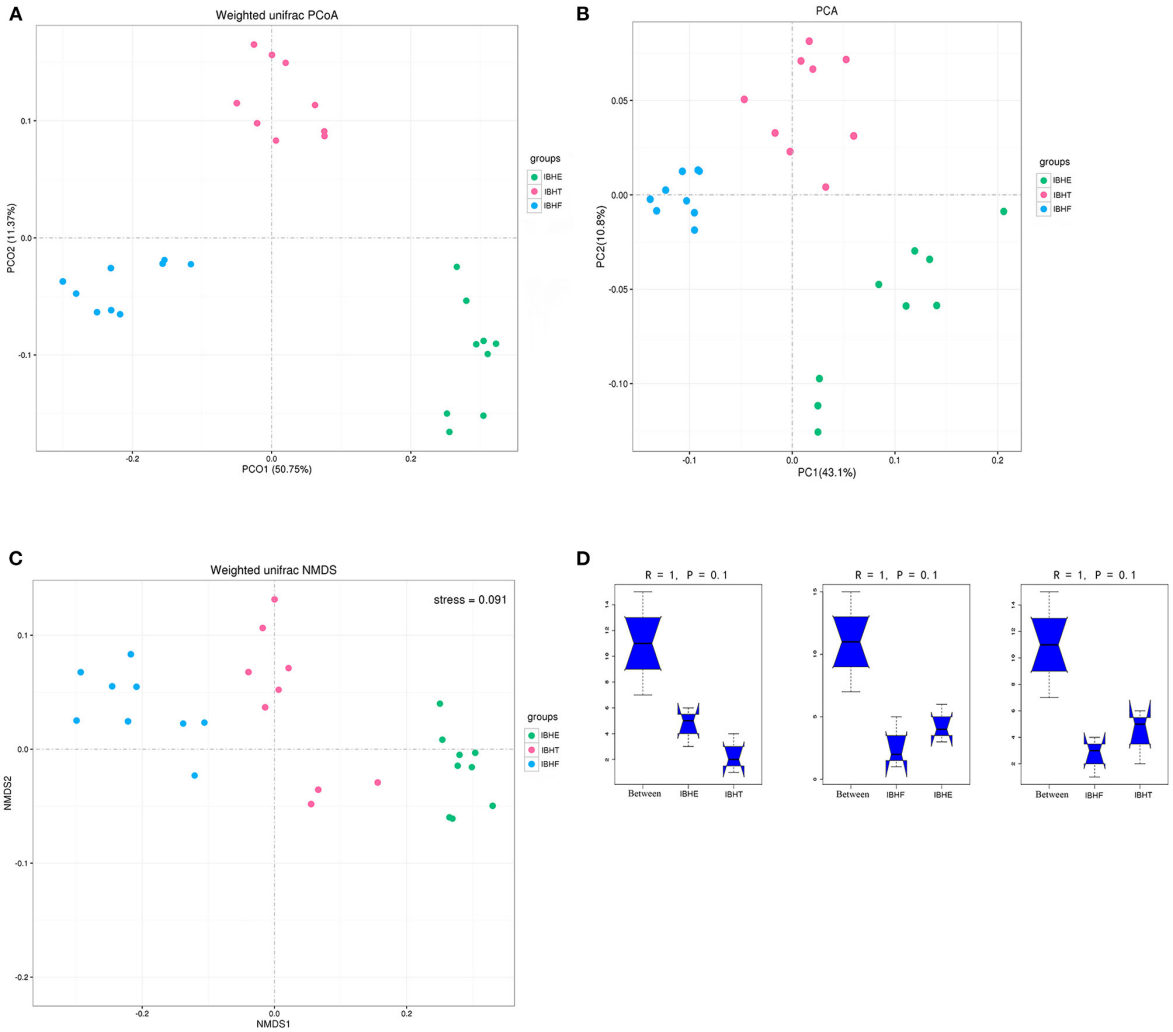
β-Diversity analysis of samples at 8, 20, and 50 weeks. **(A)** Weighted uniFrac principal coordinates analysis (PCoA) of samples at 8, 20, and 50 weeks. IBHE, IBHT, and IBHF refer to “ISA Brown Hens-8 weeks,” “ISA Brown Hens-20 weeks,” and “ISA Brown Hens-50 weeks,” respectively. Dots of different colors represent samples in different growth periods. The closer the distance between the two points, the smaller the difference of community composition. The bottom right corner of the picture (green) was IBHE, the top right corner (pink) was IBHT, and the left corner (blue) was IBHF. **(B)** Weighted uniFrac principal component analysis (PCA) of samples at 8, 20, and 50 weeks. In PCoA and PCA plots, abscissa and ordinate represent the first and second principal component explaining the greatest proportion of variances in the communities, respectively. **(C)** non-metric multidimensional scaling (NMDS) of samples at 8, 20, and 50 weeks. The stress value is used to estimate the accuracy of the model. The closer the stress value approaches zero, the better the model effect is. The model with stress value < 0.1 can be accepted. **(D)** Anosim analysis of samples at 8, 20, and 50 weeks. The abscissa represents the comparison among all samples (between) and the comparison within each group, and the ordinate represents the distance between samples. The range of rank (*R*) is −1 to 1. *R* > 0 indicates that the difference between groups was greater than that within groups. *P*-value > 0.05 indicates no statistical significance.

For the β-diversity indices, the PCA (Figure 2A) and PCoA (Figure 2B) showed that the samples were separated in the first principal component. NMDS showed that the stress = 0.091 < 0.1, indicating that the accuracy of the model was good (Figure 2C). In addition, Anosim test showed that R = 1 > 0, indicating that the difference of microorganisms between groups was greater than that within groups; however, *P* = 0.1 > 0.05 represented that there was no significant difference in the pairwise comparison of three periods (Figure 2D). On the whole, the samples were clustered by different growth periods, but there was no significant difference between different periods (*P* > 0.05).

### Gut Microbial Composition in Different Growth Periods

**Figure 3:**
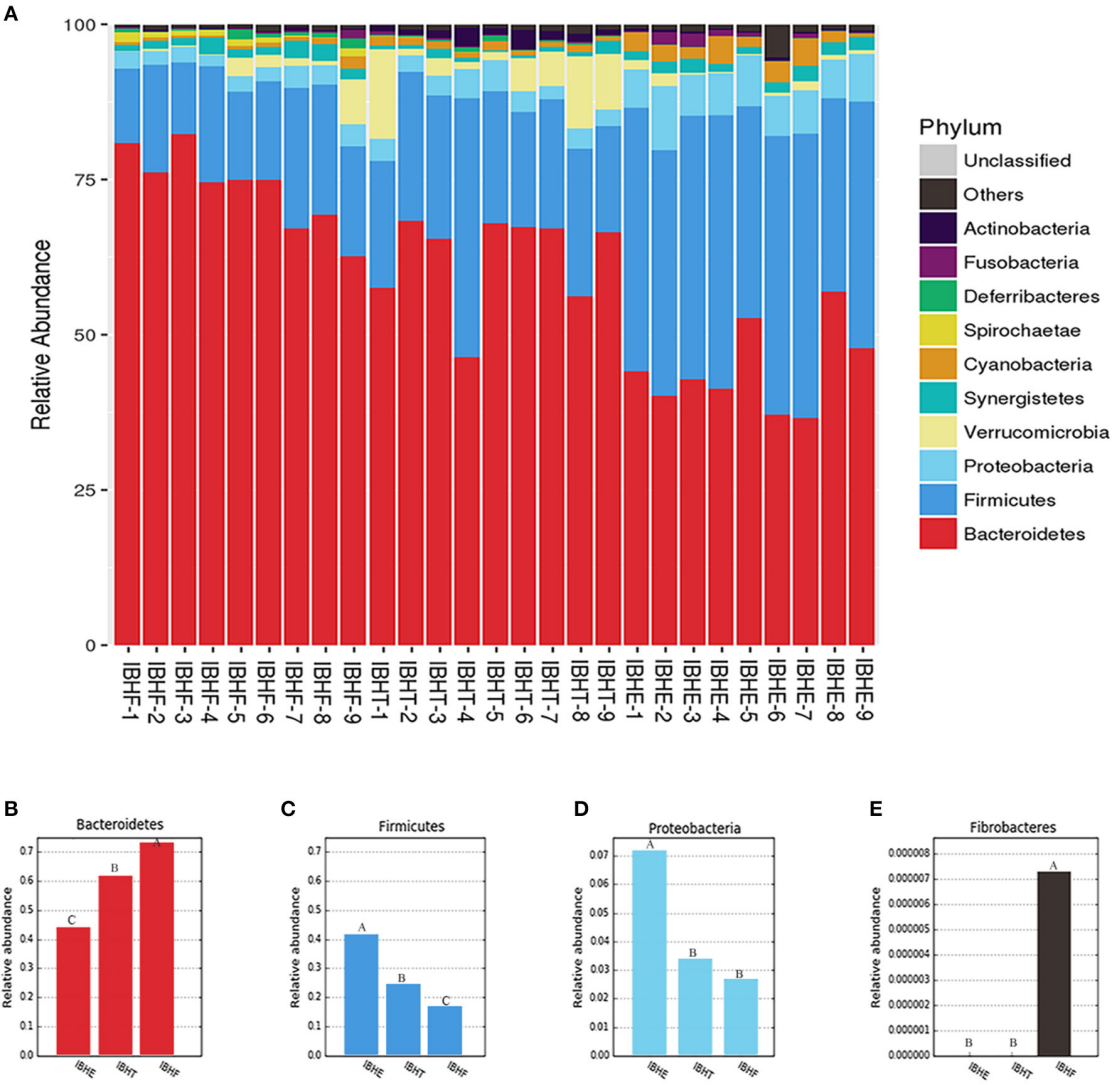
Taxonomy stack distribution and abundance histograms of the dominant bacteria phyla of chickens in different growth periods. **(A)** Taxonomy stack distribution of the dominant phyla at 8, 20, and 50 weeks. IBHE, IBHT, and IBHF refer to “ISA Brown Hens-8 weeks,” “ISA Brown Hens-20 weeks,” and “ISA Brown Hens-50 weeks,” respectively. The top 10 dominant bacteria during different periods were classified. A distinctive color histogram represents the relative distribution of these most dominant bacteria. The names of distinct levels of phyla are shown on the right of figures, and the names of samples are under the figure. **(B–E)** Abundance histograms of some significant dominant phyla at 8, 20, and 50 weeks. Phyla with different superscript letters mean significant differences (*P* < 0.05) between groups, and different superscript letters mean extremely significant differences (*P* < 0.01).

At the phylum level (Figure 3A), the dominant phyla of all samples were both Firmicutes and Bacteroidetes. The abundance of “generalist” Bacteroidetes (44.40, 62.55, and 73.65%) (Figure 3B) significantly increased at 8, 20, and 50 weeks, respectively (*P* < 0.01), but Firmicutes (40.47%, 23.41, and 16.76%) (Figure 3C) and Proteobacteria (7.27, 3.37, and 2.70%) (Figure 3D) significantly decreased with age (*P* < 0.01). Special cellulose-degradation phylum Fibrobacteres was only found at 50 weeks (*P* < 0.01) (Figure 3E).

**Figure 4:**
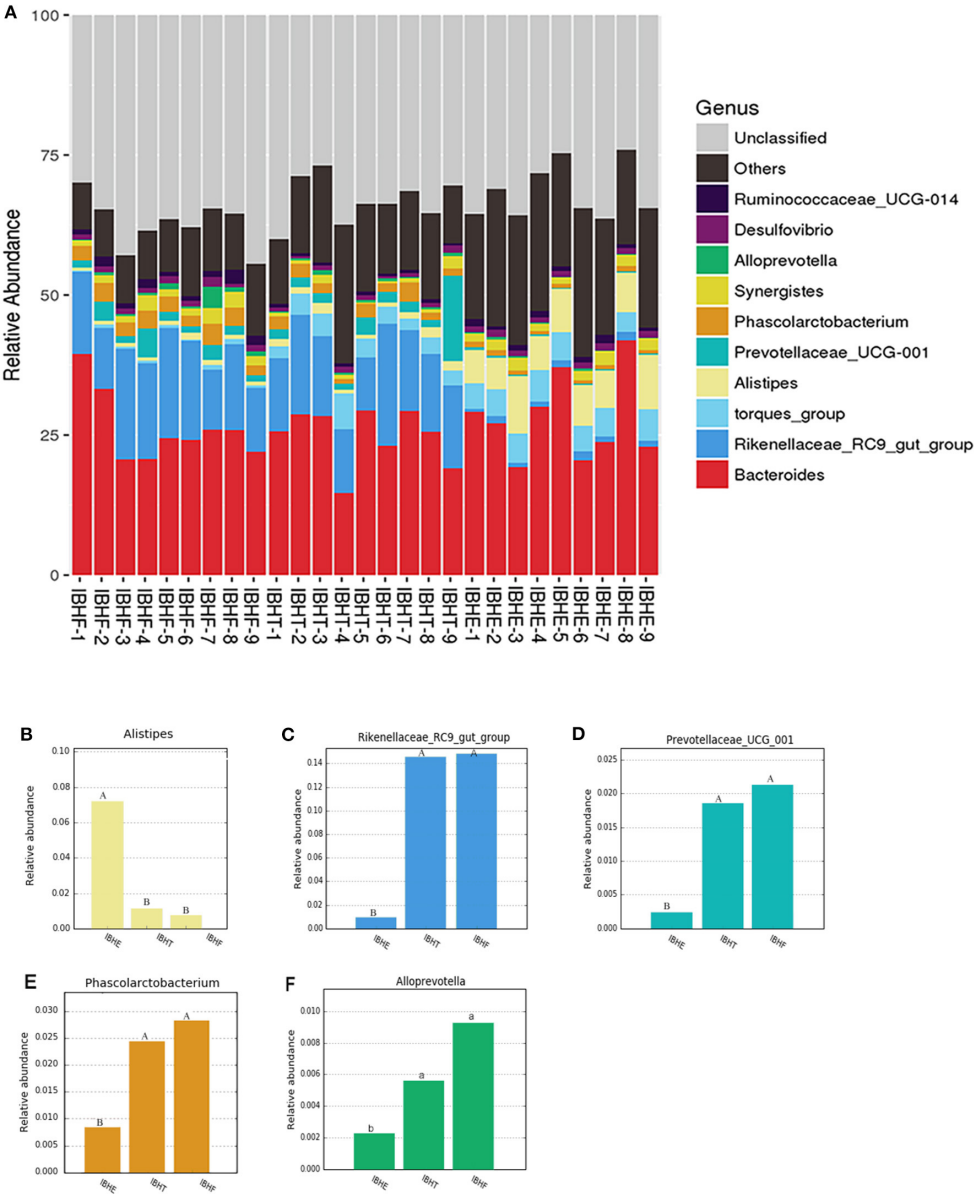
Taxonomy stack distribution and abundance histograms of the dominant bacteria genera of chickens in different growth periods. **(A)** Taxonomy stack distribution of the dominant genera at 8, 20, and 50 weeks. IBHE, IBHT, and IBHF refer to “ISA Brown Hens-8 weeks,” “ISA Brown Hens-20 weeks,” and “ISA Brown Hens-50 weeks,” respectively. The top 10 dominant bacteria genera during different periods were classified. A distinctive color histogram represents the relative distribution of these most dominant bacteria. The names of distinct levels of genera are shown on the right of the figures, and the names of the samples are under the figure. **(B–F)** Abundance histograms of some significant dominant genera at 8, 20, and 50 weeks. Genera with different superscript letters mean significant differences (*P* < 0.05) between groups, and different superscript letters mean extremely significant differences (*P* < 0.01).

**Figure 5:**
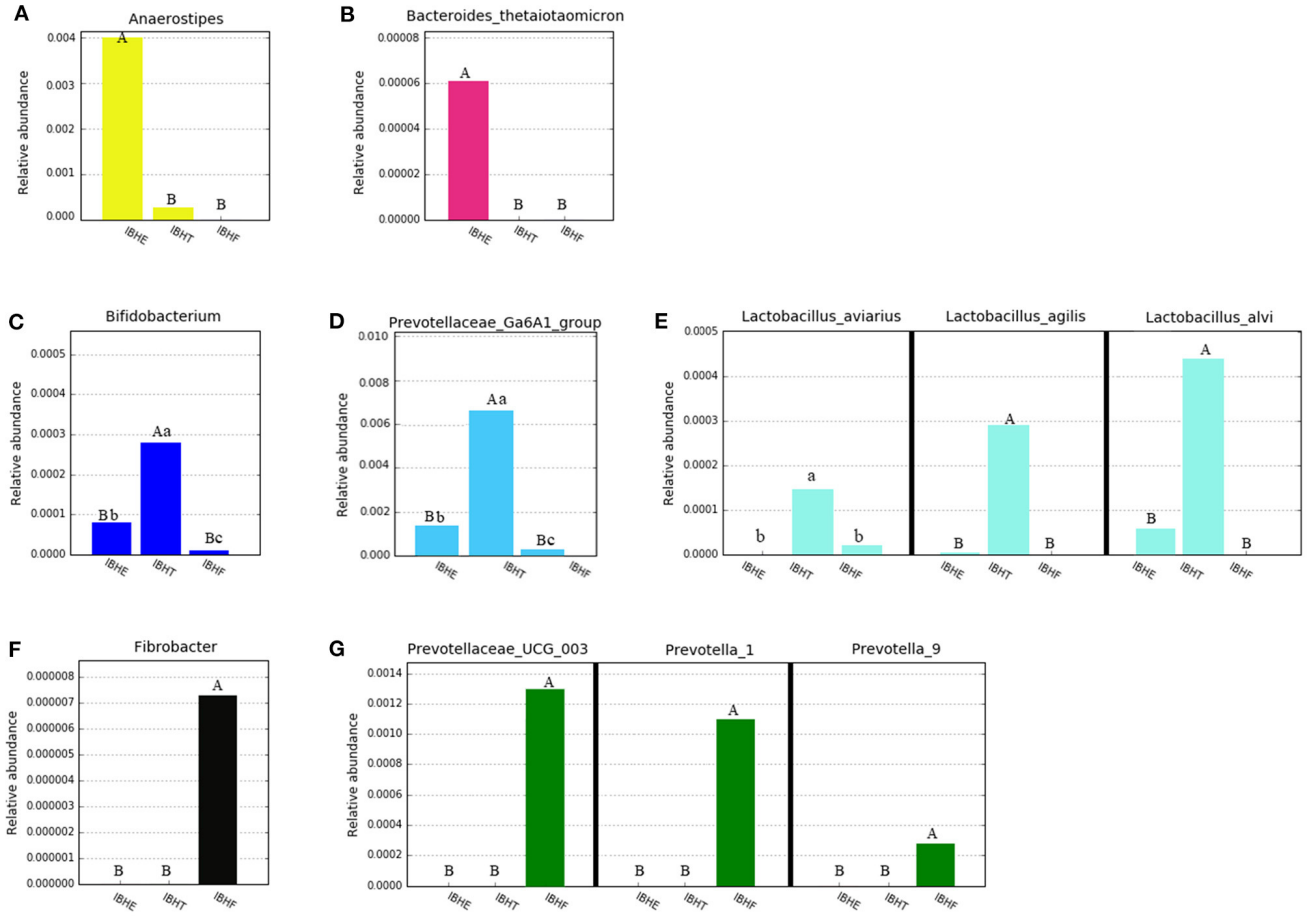
Abundance histograms of some low-abundance dominant bacteria of chickens in different growth periods. **(A,B)** Abundance histograms of significant dominant short-chain fatty acid (SCFA)-producing bacteria *Anaerostipes* and fiber-degradation bacteria species *Bacteroides thetaiotaomicron* at 8 weeks. **(C–E)** Abundance histograms of significant dominant fiber-degradation bacteria genera *Prevotellae_Ga6A1_group* and *Bifidobacterium* and SCFA-producing species of *Lactobacillus* at 20 weeks. **(F,G)** Abundance histograms of dominant fiber-degradation bacteria genera at 50 weeks. Bacteria with different superscript letters mean significant differences (*P* < 0.05) between groups, and different superscript letters mean extremely significant differences (*P* < 0.01).

At the genus level (Figure 4A), the dominant genus of all samples was *Bacteroides* (27.97, 24.84, and 26.29%) (*P* > 0.05). The dominant bacteria at 8 weeks included the bile-tolerant bacterium *Alistipes* (7.38%) (Figure 4B) and low-abundance butyrate-producing genus *Anaerostipes* (0.4%) (Figure 5A) compared with 20 weeks (1.23%) (0.033) and 50 weeks (0.76%) (0.00021) (*P* < 0.01), respectively. In addition, *Bacteroides thetaiotaomicron* (0.006%), which is one of the best fiber-degrading species, was only detected at 8 weeks (Figure 5B). The dominant bacteria genera at 20 and 50 weeks included *Rikenellaceae_RC9_gut_group* (14.56 and 15.23%) (*P* < 0.01) (Figure 4C), propionate-producing genus *Phascolarctobacterium* (1.92 and 2.76%) (*P* < 0.01) (Figure 4E), fiber-degradation bacteria *Prevotellae_UCG_001* (1.81 and 2.13%) (*P* < 0.01) (Figure 4D), and *Alloprevotella* (0.56 and 0.92%) (*P* < 0.05) (Figure 4F) compared with those at 8 weeks (1.09, 0.80, 0.26, and 0.21%), respectively.

The dominant bacteria at 20 weeks also included low-abundance fiber-degradation and acetate-producing genus *Bifidobacterium* (0.028%) (Figure 5C) and potential fiber-degradation bacteria *Prevotellae_Ga6A1_group* (0.62%) (Figure 5D) compared with those at 8 and 50 weeks. *Bifidobacterium* was also a famous probiotic genus. The dominant bacteria at 20 weeks also included some lactate-producing species such as *Lactobacillus aviaries* (0.014%), *Lactobacillus agilis* (0.029%), and *Lactobacillus alvi* (0.043%) (Figure 5E). Moreover, many unique fiber-degradation bacteria including *Fibrobacter* (0.00072%), *Prevotellae_UCG_003* (0.13%), *Prevotella_1* (0.11%), and *Prevotella_9* (0.0026%) were only found at 50 weeks (Figures 5F,G).

### Functional Prediction of the Gut Microbiota in Different Growth Periods

**Figure 6:**
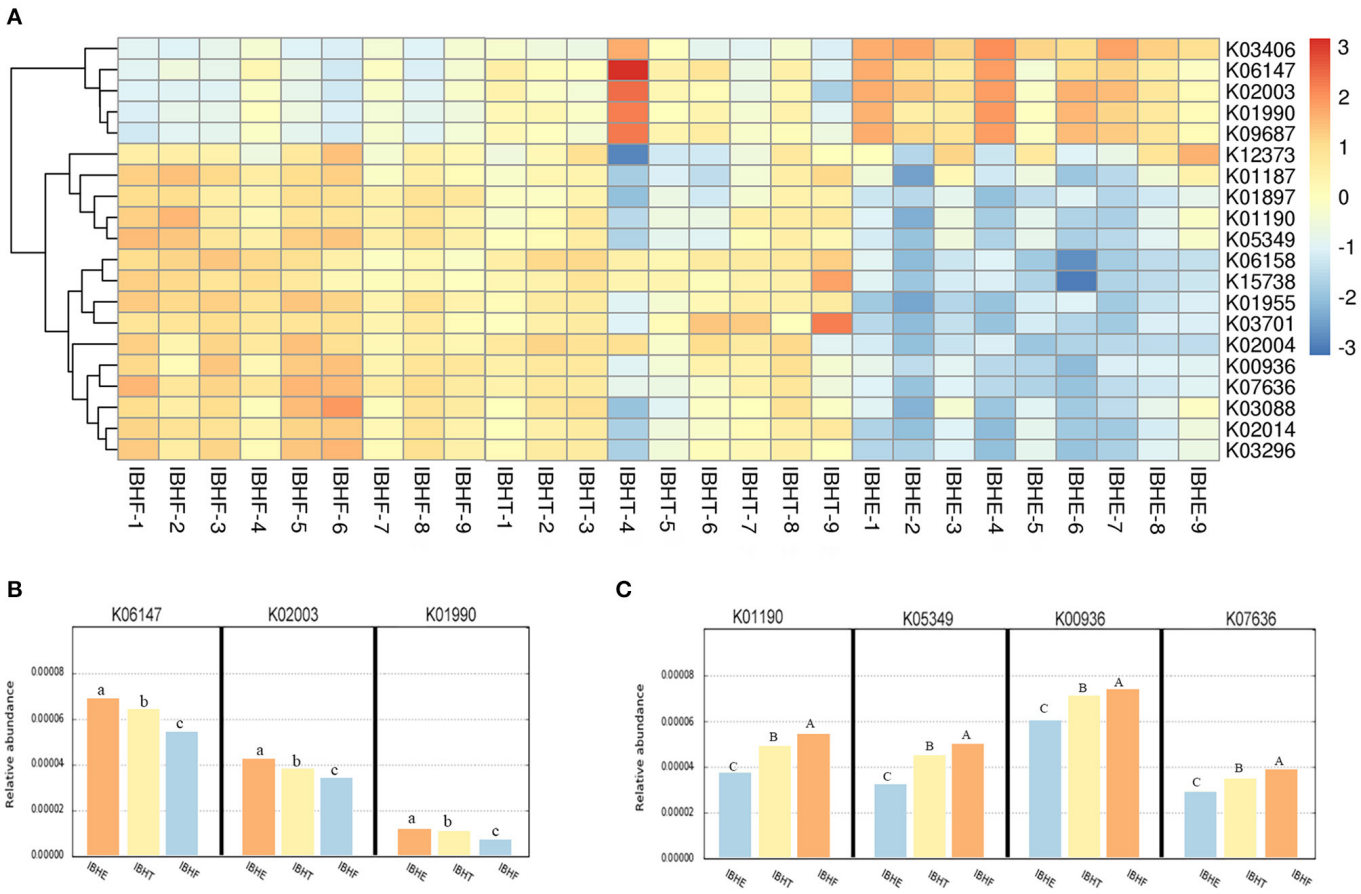
**(A)** The heatmap of functional prediction of Kyoto Encyclopedia of Genes and Genomes (KEGG) Orthology (KO) of operational taxonomic units (OTUs) in different growth periods. The color gradient of heatmap was from red to yellow to blue, which indicates that the KO abundance predicted by OTU in each sample decreased gradually. The redder the color, the more bacteria performed this function. The bluer the color, the lesser bacteria performed this function. IBHE, IBHT, and IBHF refer to “ISA Brown Hens-8 weeks,” “ISA Brown Hens-20 weeks,” and “ISA Brown Hens-50 weeks,” respectively. **(B)** The abundance histograms of significantly reduced KO of OTUs from 8 to 20 and 50 weeks. **(C)** The abundance histograms of significantly increased KO of OTUs from 8 to 20 and 50 weeks. KO with different superscript letters means significant differences (*P* < 0.05) between groups, and different superscript letters mean extremely significant differences (*P* < 0.01).

The KO of OTUs was further predicted (Figure 6A). The heatmap showed that ATP-binding cassette (ABC) transports including K06147 (0.0066, 0.0063, and 0.0056%), K02003 (0.0042, 0.0039, and 0.0036%), and K01990 (0.0031, 0.0030, and 0.0027%) significantly decreased at 8, 20, and 50 weeks (*P* < 0.05) (Figure 6B), respectively. In contrast, β-galactosidase K01190 (0.0039, 0.0050, and 0.0056%), β-glucosidase K05349 (0.0034, 0.0042, and 0.0051%), and two-component system (TCS) including K00936 (0.0060, 0.0071, and 0.0075%) and K07636 (0.0029, 0.0035, and 0.0039%) significantly increased with age (*P* < 0.01) (Figure 6C).

**Figure 7:**
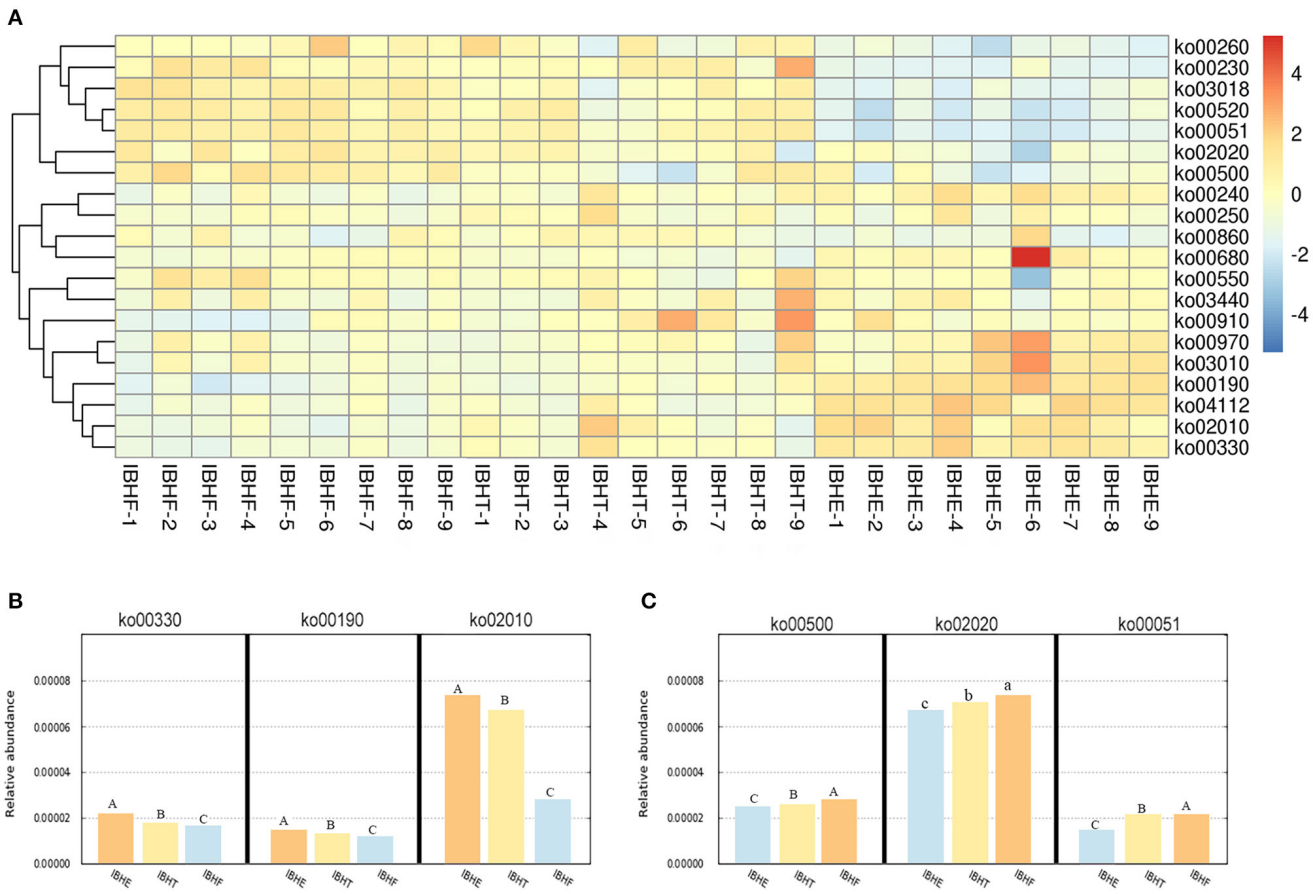
**(A)** The heatmap of functional prediction of enriched metabolic pathways (ko) of OTUs in different growth periods. The color gradient of heatmap was from red to yellow to blue, which indicates that the metabolic pathway abundance predicted by OTUs in each sample decreased gradually. The redder the color, the more bacteria performed this function. The bluer the color, the lesser bacteria performed this function. IBHE, IBHT, and IBHF refer to “ISA Brown Hens-8 weeks,” “ISA Brown Hens-20 weeks,” and “ISA Brown Hens-50 weeks,” respectively. **(B)** The abundance histograms of significantly increased metabolic pathways of OTUs from 8 to 20 and 50 weeks. **(C)** The abundance histograms of significantly reduced metabolic pathways of OTUs from 8 to 20 and 50 weeks. Metabolic pathways with different superscript letters mean significant differences (*P* < 0.05) between groups, and different superscript letters mean extremely significant differences (*P* < 0.01).

Then, the enriched metabolic pathways of OTUs were predicted (Figure 7A). Amino acid metabolism pathways ko00330 (0.021, 0.019, and 0.018%), the energy metabolism pathway ko00190 (0.017, 0.016, and 0.015%), and the membrane transport of ABC transporters ko02010 (0.075, 0.066, and 0.029%) significantly decreased at 8, 20, and 50 weeks (*P* < 0.01) (Figure 7B), respectively. In contract, carbohydrate metabolism pathways ko00500 (0.026, 0.027, and 0.029%) and ko00051 (0.017, 0.021, and 0.021%) (*P* < 0.01) and the TCS pathway ko02020 (0.068, 0.070, and 0.072%) (*P* < 0.05) significantly increased with age. In addition, the carbohydrate metabolism pathway ko00051 (0.021 and 0.021%) was enriched at 20 and 50 weeks compared with 8 weeks (0.017%) (*P* < 0.01) (Figure 7C).

### Concentration of Short-Chain Fatty Acids in Different Growth Periods

Next, the concentration of gut microbial metabolites SCFAs was measured (Table 4). The concentration of SCFAs at 20 weeks was higher than that at 8 weeks (*P* < 0.01). Interestingly, although the microbial diversity at 50 weeks was the highest, the concentration of SCFAs at 20 weeks was also almost twice as high as that at 50 weeks (*P* < 0.01).

**Table 4:** The concentration of short-chain fatty acids (SCFAs) (mmol/L) of chickens in different growth periods.

**SCFA**	**8 weeks**	**20 weeks**	**50 weeks**	**SEM**	***P*-value**
Acetate	2.42^Bc^	6.12^Aa^	3.71^Bb^	0.32	0.006
Propionate	0.62^Bb^	1.91^Aa^	0.99^Bb^	0.06	0.009
Butyrate	0.19^Bb^	0.60^Aa^	0.25^Bb^	0.021	0.005

*Means within a row lacking a common lowercase superscript letter mean significant differences with a P-value < 0.05, and means within a row lacking a common uppercase superscript letter mean extremely significant differences with a P-value < 0.01. Data are expressed as the means and pooled standard error of the mean (SEM)*.

## Discussion

### Changes of Microbial Diversity in Different Growth Periods

The gastrointestinal tract of newly hatched chickens immediately has microbial colonization (42). With the growth of age, the cecal microorganisms form a complex community (43). In this experiment, the numbers of OTUs and gut microbial α-diversity including ACE and Chao1 of chickens significantly also increased with age. The larger the value of ACE and Chao1, the higher the community richness. This is consistent with previous reports that increasing taxonomic richness and diversity were observed in chickens through time (9). The α-diversity of the gut microbiota of pigs also increased with age (21). In this experiment, the β-diversity of the samples was clustered by different growth periods, but NMDS analysis showed the difference between different periods was not great. It was consistent with a report that NMDS showed that 0- to 42-day-old chicken gut microbiota could be clustered according to different ages, but microbial clusters became quite similar after 28 days (11).

### Changes of Gut Microbiota Composition in Different Growth Periods

At the level of phyla, the predominant phyla were Firmicutes and Bacteroidetes throughout the period in this experiment, which was consistent with previous studies (6). In addition, the relative abundance of Firmicutes was higher than that of Bacteroidetes at 8 weeks in this study. It was also reported that there were around 37% Firmicutes and 10% Bacteroidetes in 8-week-old chickens (1). Firmicutes species are regarded as “specialists” for storage plant polysaccharides (starch and fructose) and oligosaccharides. In contrast, Bacteroidetes is a kind of “generalist” that degrades dietary fiber. It can utilize more a wide range of plant polysaccharides than does Firmicutes (19). In this experiment, Bacteroidetes increased from 8 to 50 weeks. This was supported by studies that reported the gradual increase of Bacteroidetes at the expense of Firmicutes during chicken rearing and egg production (18). Members of Bacteroidetes were present mainly in adult hens (44).

At the genus level, the composition of the gut microbiota in different growth periods was also different in this experiment. The dominant bacteria genus at 8 weeks did not include fiber-degradation bacteria but included the bile-tolerant bacterium *Alistipes* compared with that at 20 and 50 weeks. This was maybe attributed to a high-fat and low-fiber diet (45). *Alistipes* specially increased in the persons who consumed animal-based diet instead of plant-based diet (46). In contrast, fiber-degradation bacteria genus *Prevotellae_UCG_001* and *Alloprevotella* obviously increased at 20 and 50 weeks compared with 8 weeks. For 20 weeks with a high-fiber diet, this because the fiber content of it was higher than that at 8 weeks, resulting in increased fiber-degradation bacteria and *Prevotellae_Ga6A1_group* and *Bifidobacterium*. Research on human showed that the abundance of *Prevotella* is enriched in a fiber diet (47) and has a strong ability to utilize fiber (48). *Prevotella* gradually became the most diverse and predominant genus with the increase of dietary fiber and age in pigs (21). The research showed that increasing dietary fiber could increase the abundance of *Bifidobacterium* (49). For 50 weeks with a low-fiber diet, the feed intake was greater than that at 8 weeks, resulting in more dietary fiber being ingested. This may also help to explain why fiber-degradation bacteria *Prevotella_1* and *Prevotella_9* and excellent special cellulose-degradation genus *Fibrobacter* were only detected at 50 weeks in this experiment. Notably, as far as we know, it seems that *Fibrobacter* has not been reported in chickens. *Fibrobacter* was once believed to only exist in mammalian intestines (50), and it was first reported in the cecum of birds (ostrich) in 2010 (51).

### Function Annotation of Gut Microbiota in Different Growth Periods

Software packages such as Tax4Fun can be used to predict the functional profiles of OTUs using 16S rRNA gene sequences based on the SILVA rRNA database (52). In this experiment, functional annotation of the gut microbiota showed that ABC transporters decreased at 8, 20, and 50 weeks. This was related to the dominant phylum, which was Firmicutes, decreasing during this period. Firmicutes has gram-positive polysaccharide utilization loci (PULs) (gpPULs), which encode ABC transporters and other transporters to introduce small sugar into the periplasm for processing (15, 53). ABC transporter is a type of transport ATPase on the bacterial plasma membrane, and it transfers glucose to the other side of the membrane through the change of conformation. Consistently, the energy metabolism pathway and amino acid metabolism pathway were rich at 8 weeks in this study. Research also reported that the microbiota in the hindgut of 42-day-old chickens were enriched in amino acids metabolism and energy metabolism according to KEGG functional analysis (11).

In contrast, functional annotation of OTUs included a TCS; and β-glycosidase, carbohydrate metabolism pathway, and TCS pathway increased at 8, 20, and 50 weeks. This was related to the fact that the abundance of Bacteroidetes increased with age. Contrary to the gpPULs of Firmicutes, Bacteroidetes can utilize a series of plant-derived dietary polysaccharides via unique PUL (54, 55). PUL has been identified in all members of Bacteroidetes such as *B. thetaiotaomicron* and *Bacteroides ovatus* (19). PUL encodes a hybrid TCS, extracellular glycoside hydrolase such as β-glycosidase (56), and other enzymes to degrade dietary fiber by cleaving glycosidic bonds (19). Consistently, carbohydrate metabolism pathway was increased with age in this experiment.

### The Relation Between Short-Chain Fatty Acids and Microorganisms in Different Growth Periods

SCFAs are the major fermented metabolites of dietary fiber for SCFA-producing bacteria; primarily acetate, propionate, and butyrate account for 90–95% (57). SCFAs are very important for the growth and health of host.

In this study, the concentration of SCFAs at 20 weeks was higher than that at 8 weeks. This may be attributed to the level of fiber increase, and the numbers of some dominant fiber-degradation bacteria and SCFA-producing bacteria were more in 20 weeks. The dominant fiber-degradation bacteria at 20 weeks such as *Prevotellae_UCG_001* and *Alloprevotella* broke down dietary fiber into more monosaccharides, and then dominant SCFA-producing bacteria *Phascolarctobacterium, Bifidobacterium*, and *Lactobacillus* fermented monosaccharides into more SCFAs than those at 8 weeks. *Phascolarctobacterium* can ferment monosaccharide into propionate (58), and *Bifidobacterium* produces acetate using “bifid-shunt” (59). Members of *Lactobacillus* use dietary fiber via ABC transporters to produce acetate (60), which can be further fermented into butyrate by some butyrate-producing bacteria (61).

In addition, the concentration of SCFAs at 20 weeks was also greater than that at 50 weeks in this experiment. Given that the relative abundance of the dominant SCFA-producing bacteria showed no great difference between them, we speculated that it was more likely that although the microbial α-diversity including ACE and Chao1 at 50 weeks was higher than that at 20 weeks, most of the bacteria were not fiber-degradation bacteria or SCFA-producing bacteria; this means that more bacteria compete for limited glucose as the carbon for growth and that less glucose was fermented into SCFAs by few SCFA-producing bacteria. This may also help to explain why obese people have less microbial diversity but have more SCFAs than lean people (62).

## Conclusions

The diversity, composition, and function of the gut microbiota of chickens were distinct in different growth periods. The relative abundance of the bile-acid resistant bacteria *Alistipes* was higher at 8 weeks compared with 20 and 50 weeks. Fiber-degradation bacteria *Prevotellae_UCG_001* and *Alloprevotella* and SCFA-producing bacteria *Phascolarctobacterium* increased at 20 and 50 weeks compared with 8 weeks. In addition, ABC transporters decreased from 8 to 50 weeks; it might because the abundance of Firmicutes—which includes gpPULs—decreased with age. In contrast, the TCS, glucosidase, and carbohydrate metabolism pathway gradually increased from 8 to 50 weeks, because the abundance of Bacteroidetes—which includes PULs—increased with age. The concentration of SCFAs in the cecum at 20 weeks was higher than 8 and 50 weeks.

## Data Availability Statement

The datasets presented in this study can be found in online repositories. The names of the repository/repositories and accession number(s) can be found below: NCBI SRA; PRJNA701972.

## Ethics Statement

The animal study was reviewed and approved by Shanxi Agricultural University Animal Experiment Ethics Committee (the license number: SXAU-EAW-2017-002Chi.001).

## Author Contributions

YY: conceptualization, resources, supervision, and funding acquisition. LH: methodology and visualization. BS and LH: formal analysis, investigation, and data curation. BS: writing—original draft preparation and review and editing. YY and BS: project administration. All authors contributed to the article and approved the submitted version.

## Conflict of Interest

The authors declare that the research was conducted in the absence of any commercial or financial relationships that could be construed as a potential conflict of interest.
